# Combined Administration of Bisphosphonates, Chemotherapeutic Agents, and/or Targeted Drugs Increases the Risk for Stage 3 Medication-Related Osteonecrosis of the Jaw: A 4-Year Retrospective Study

**DOI:** 10.1155/2020/5847429

**Published:** 2020-10-15

**Authors:** Yuqiong Zhou, Yejia Yu, Yueqi Shi, Mengyu Li, Chi Yang, Shaoyi Wang

**Affiliations:** National Clinical Research Center for Oral Disease, Shanghai Key Laboratory of Stomatology and Shanghai Research Institute of Stomatology, Department of Oral Surgery, Shanghai Ninth People's Hospital, College of Stomatology, Shanghai Jiao Tong University School of Medicine, 200011, China

## Abstract

**Objectives:**

Patients with stage 3 medication-related osteonecrosis of the jaw (MRONJ) suffer from severe complications. Chemotherapeutic agents and targeted drugs are considered to be associated with the development of MRONJ. However, little is known regarding the association of those agents with stage 3 MRONJ. The purpose of this study is to analyze the comprehensive medication history of patients with advanced-stage MRONJ (stage 2 and stage 3) and evaluate the possible risk factors for stage 3 MRONJ. *Patients and Methods*. Sixty patients with advanced-stage MRONJ were involved in this retrospective study. Patients with developmental maxillofacial anomalies, previous radiation in the head and neck areas, and jaw bone tumors were excluded from the study. All patients were divided into two groups by their MRONJ stage (stage 2 or stage 3). Demographic and clinical characteristics, comprehensive medication data (bisphosphonates, chemotherapeutic agents, targeted drugs, and immunosuppressive agents), and results of serological biomarkers were recorded and compared between two groups. Univariate and multivariate logistic regressions were performed by SPSS 25.0 for evaluating risk factors of stage 3 MRONJ.

**Results:**

Our results indicate that chemotherapy (adjusted OR = 3.43; 95% CI: 1.03 to 11.38), targeted drugs (adjusted OR = 3.69; 95% CI: 1.06 to 12.80), and maxillary lesions (adjusted OR = 4.26; 95% CI: 1.19 to 15.23) increase the risk of stage 3 MRONJ.

**Conclusion:**

The outcome of this study justifies that chemotherapeutic agents and targeted drugs are probably risk factors for stage 3 MRONJ. In addition, the osteonecrosis in maxilla is more easily to develop into stage 3 MRONJ. Intense clinical observation is recommended in MRONJ patients with maxillary osteonecrosis and in those who concurrently administered bisphosphonates, chemotherapeutic agents, and/or targeted drugs. This trial is registered with ChiCTR2000032428.

## 1. Introduction

Medication-related osteonecrosis of the jaw (MRONJ) is a rare complication that is defined as exposed bone or bone that can be probed through fistulas in the maxillofacial region over 8 weeks [1]. Patients who have special medication history and no radiation of head and neck can be diagnosed as MRONJ. The use of bone-modifying agents (BMAs), including bisphosphonates (BPs) [2] and denosumab [3], and angiogenic inhibitor agents is considered responsible for the development of MRONJ. The antiangiogenesis substances include antivascular endothelial growth factor (VEGF), tyrosine kinase inhibitors, mTOR inhibitors, human monoclonal antibodies, and thalidomide [4]. Additionally, chemotherapy, BRAF inhibitors, TNF inhibitors, and immune checkpoint inhibitors are found to be related to MRONJ probably [5, 6].

Patients with advanced-stage MRONJ (stage 2 and stage 3) complain of pain, swelling, purulence, numbness, significant ozostomia, and so on. MRONJ patients with stage 3 who suffer from facial fistula, oral nasal, maxillary sinus communication, or pathological fracture tend to be more serious than patients with stage 2 [7]. Additionally, the management of two stages is diverse. On the basis of conservative measures, patients with stage 2 MRONJ could undergo conservative surgery, while invasive surgery might be considered for patients with stage 3 MRONJ [7]. Nevertheless, due to the long-term accumulation of BMAs or antiangiogenic agents, the damaged jaw in MRONJ patients has poor healing ability, which increases the recurrence rate after surgical treatment. Thus, patients with stage 3 MRONJ have a lower quality of life and poor prognosis [8].

Studies demonstrated that the dosage, routes (IV or oral), and duration of BMAs are implicated in the incurrence [2, 9, 10]. Other risk factors, such as dental-alveolar surgery, denture use, periodontitis, tobacco use, and diabetes, also play a critical role in the development of MRONJ [11–13]. The use of chemotherapeutic agents and targeted drugs, as regular antineoplastic therapy, is considered to be associated with the development of MRONJ [14, 15]. However, little is known regarding whether those agents are the risk indicators of stage 3 MRONJ. Oteri et al. [16] reported that chemotherapy could worsen the clinical manifestation of MRONJ. Nevertheless, the conclusion was limited due to the absence of data from a case and control study and insufficient statistical evidence.

The purpose of this retrospective study is to investigate the potential factors which can increase the risk of stage 3 MRONJ and influence the severity of MRONJ. To our knowledge, this is the first study to report the medication use of advanced-stage MRONJ patients in detail and evaluate the risk factors for stage 3 MRONJ.

## 2. Patients and Methods

### 2.1. Patients and Groups

Between January 2016 and January 2019, a 4-year retrospective study was conducted on patients with stage 2 and stage 3 MRONJ from the Department of Oral Surgery at the Ninth People's Hospital. Patients who were diagnosed with bisphosphonate-related osteonecrosis of the jaw were involved in this study. Patients with developmental maxillofacial anomalies, previous radiation in the head and neck areas, and jaw bone tumors were excluded from the study. Conservative management and/or operative management were conducted in enrolled patients. MRONJ stage shift or stability might have occurred in patients during treatment. The most serious stage ever discovered in the follow-up before conducting surgical therapy was recorded as the basis of grouping. All MRONJ stages were identified by two experienced oral surgeons according to the medical records of the patients. The relevant MRONJ stage classifications, as defined by the 2019 Multinational Association of Supportive Care in Cancer/International Society of Oral Oncology/American Society of Clinical Oncology (MASCC/ISOO/ASCO) Clinical Practice Guideline [7], are listed in Table 1. With regard to the grouping, patients with stage 2 MRONJ were assigned to group A, while patients with stage 3 MRONJ were assigned to group B. This study was conducted with the understanding and written consent of every patient and in accordance with the tenets of the Declaration of Helsinki. This study was independently reviewed and received approval from the institutional ethical committee of the Ninth People's Hospital of Shanghai Jiao Tong University School of Medicine (SH9H-2020-T37-1).

### 2.2. Variables and Data Collection

All variables and data were collected by the same researcher who did not know the group. A standardized and comprehensive history was obtained from each patient. The clinical features, radiographic data, and results of laboratory examinations of patients at the most serious MRONJ stage were applied.

Demographic and clinical features were collected including sex, age, smoking habit, cancer type, osteoporosis, basic systemic diseases, dental extraction, denture use, periodontal disease, and lesion site. The medication history was recorded in detail, including the type and duration of cumulative bisphosphonates, chemotherapy, targeted agents, and immunosuppressive agents. Immunosuppressive agents comprise thalidomide, anti-PD-1, and glucocorticoids (GCs) which were administrated over 6 months. The time from initially using bisphosphonates to the onset of MRONJ was recorded as the TTO reported by Fung et al. [17]. For the patients who had stopped using bisphosphonates before the onset of MRONJ, the discontinuance time (dTTO) was calculated as well.

Radiographic data, including panoramic radiography and computed tomography (CT), was reviewed to define the MRONJ stage of the patients. CT scans that revealed the most serious stage were used to determine the boundary of lesion and complications such as pathological fracture. All CT scans were taken by the same technician using the same device (Planmeca Oy, Helsinki, Finland) in the Oral Radiology Department at the Ninth People's Hospital. For group B, the invasion of the maxillary sinus, nasal base, mandibular canal, inferior board of the mandible, and ramus of the mandible as well as the occurrence of facial fistula and pathological fracture were investigated. Some laboratory blood tests, including *β*-CTX, propeptide of type I procollagen (PINP), osteocalcin (OC), calcitonin, parathyroid hormone (PTH), C-reactive protein (CRP), 25-hydroxy vitamin D, Ca, Mg, phosphorus (P), and alkaline phosphatase (ALP), were examined by the Department of Laboratory Medicine at the Ninth People's Hospital.

### 2.3. Statistical Analysis

SPSS 25.0 (SPSS Inc., Chicago, IL) was used for statistical analysis. All continuous variables were checked for normal distribution via the Shapiro-Wilk test for normality. As the normality assumption was met, the mean and standard deviation of continuous variables were calculated. The independent sample *t*-test was used to compare the statistical difference of continuous variables. Categorical variables were reported as the number or percentage with the characteristic of interest. Pearson's *χ*^2^ test was used for between-group comparisons of categorical variables. Probabilities of less than 0.05 were accepted as significant. Crude and adjusted odds ratios (ORs) as well as corresponding 95% confidence intervals (CIs) were estimated by univariate and multivariate logistic regressions.

## 3. Results

### 3.1. Demographic and Clinical Features

**Figure 1:**
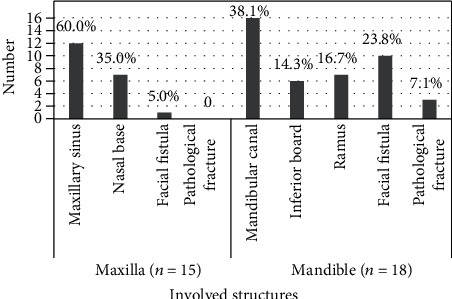
Distribution of different complications in patients with stage 3 MRONJ. Among the patients with maxillary lesions, the involvement of the maxillary sinus and nasal base and the occurrence of facial fistula and pathological fracture were calculated. Except for facial fistula and pathological fracture, the involvement of the mandibular canal, inferior board, and ramus in the mandible was recorded. The proportion of each complication was calculated, respectively.

A total of 60 patients were enrolled in this study. The median age was 70.2 (±8.0 SD; range 60-89 years), and 32 (53.3%) patients were male. Of these patients, 31 (51.7%) were in group A, and 29 (48.3%) were in group B. The comparison of demographic and clinical characteristics between two groups was recorded (Table 2). No significant differences were observed in age, sex, smoking, general diseases, local factors, endocrine drugs, immunosuppressive drugs, TTO, dTTO, and so on (Table 2). A significantly higher proportion of chemotherapeutic agents was noted in group B (*P* = 0.004). Similarly, the use of targeted drugs in group B was significantly higher than group A (*P* = 0.009). For the site of the lesion, there was a significant difference between two groups in maxillary osteonecrosis (*P* = 0.009). Moreover, the distribution of severe complications of patients in group B is presented in Figure 1. Involvement of the maxillary sinus was the most common complication in the patients with maxillary lesions (12 of 15 patients; 80.0%). Among the patients with mandible lesions, a higher proportion of mandibular canal involvement was noted (16 of 18 patients; 88.9%). Facial fistula and pathological fracture were mostly occurred in the patients with mandible lesions.

### 3.2. Medication Data and Laboratory Tests

**Figure 2:**
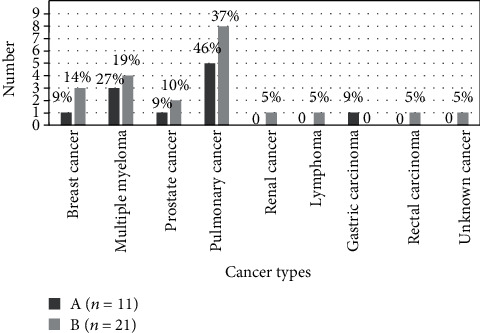
The utilization of chemotherapy in different cancer types between two groups. MRONJ patients with administration of chemotherapeutic agents in group A and group B were 11 and 21, respectively. The proportion of every cancer type (%) was the number of different cancer types of patients receiving chemotherapy divided by the total number of MRONJ patients receiving chemotherapy

Comprehensive data of medication use, including bisphosphonates, chemotherapy, targeted drugs, and immunosuppressive drugs, are recorded in Table 3. Pemetrexed was the most used chemotherapy in MRONJ patients (17 of 32 patients; 53.1%). The use of targeted drugs among advanced-stage MRONJ was distributed, and gefitinib was relatively used more frequently than other drugs (8 of 23 patients; 34.5%). However, there were no significant differences in the total duration of targeted drugs and the total time of chemotherapy between the two groups (*P* > 0.05). In addition, the use of chemotherapy in patients with different cancers between two groups is depicted in Figure 2. Of all cancer patients in this study, pulmonary cancer patients received chemotherapeutic agents at most (13 of 32 patients; 40.6%). No significant differences were found between two groups on the results of the laboratory tests (Table 4).

### 3.3. Risk Factor Analysis

Applying univariate and multivariate logistic regressions, we found that chemotherapy (OR_crude_ = 4.77, 95% CI: 1.59 to 14.30; OR_adjusted_ = 3.43, 95% CI: 1.03 to 11.38), targeted drugs (OR_crude_ = 4.22, 95% CI: 1.38 to 12.88; OR_adjusted_ = 3.69, 95% CI: 1.06 to 12.80), and maxillary lesions (OR_crude_ = 4.46, 95% CI: 1.41 to 14.11; OR_adjusted_ = 4.26, 95% CI: 1.19 to 15.23) significantly increased the risk for stage 3 MRONJ (Table 5, model 1).

Chemotherapy, targeted drugs, and maxillary lesions were inserted into the multivariate logistic regression models, and three multivariate models were used. In the first model, chemotherapy, targeted drugs, and maxillary lesions were included. All of them revealed significant ORs. Maxillary lesions proved to be associated with MRONJ progression at most (Table 5). The second model and the third model included maxillary lesions and chemotherapy or targeted drugs, respectively. Both models yielded a significant OR after adjusting for the influence of maxillary lesions, which was consistent with the result of multivariate logistic regression of the first model.

## 4. Discussion

To our knowledge, this is the first study to investigate the risk factors for stage 3 MRONJ. A wide range of risk factors for the development of MRONJ have been reported. Chemotherapy and targeted drugs are considered to be related to MRONJ. However, few studies have investigated about whether those drugs are associated with stage 3 MRONJ. Patients with stage 3 MRONJ have a lower quality of life and a poorer prognosis than patients with stage 2 MRONJ [18]. Therefore, it is meaningful to investigate the risk factors for stage 3 MRONJ. In the present study, chemotherapy, targeted drugs, and maxillary lesions were identified as the possible risk indicators for stage 3 MRONJ.

The cytotoxic effects of chemotherapy on bone metabolism and vascularization play a role in the development of MRONJ. Banfi et al. [19] found that chemotherapy had dose-dependent toxicity to the bone marrow in patients with non-Hodgkin's lymphoma and breast cancer. DeSesa et al. [20] reported that gemcitabine inhibited angiogenesis by suppressing VEGF. However, few studies have demonstrated the association of chemotherapy with stage 3 MRONJ. Bi et al. [21] reported that chemotherapeutic agents resulted in larger sequestrum and soft tissue defects in the mouse MRONJ model. Oteri et al. [16] hypothesized that the concurrent administration of chemotherapeutic agents could worsen the clinical manifestation of MRONJ. On the basis of that, we designed a case-control study and demonstrated that combined administration of bisphosphonates and chemotherapeutic agents could increase the risk for stage 3 MRONJ. Thus, for those who had a concurrent administration of BPs and chemotherapeutic agents, dentists would be aware of the risk of severe MRONJ. Of note, our analysis indicated that pulmonary cancer was the most prevalent solid tumor type in advanced-stage MRONJ patients who had received chemotherapy, which was in accordance with another study [15]. Additionally, chemotherapy-related osteonecrosis was found to be frequent in patients with multiple myeloma in our study. It might relate to the treatment regimens for multiple myeloma which contain both chemotherapeutic agents and antiangiogenic agents.

Recent reports have suggested a relatively high MRONJ risk in patients with a combined administration of bisphosphonates and targeted drugs [22, 23]. Renal cell cancer patients were one of the first at-risk populations due to enlarged administration of targeted drugs [22]. The incidence rate of MRONJ associated with targeted therapy was reported to be 0.3%-3.4% [24, 25]. Targeted drugs, including sunitinib, erlotinib, gefitinib, icotinib, and apatinib, act as tyrosine kinase inhibitors (TKIs) which work on the epidermal growth factor receptor (EGFR) or vascular endothelial growth factor (VEGF) receptor [25, 26]. In addition, human monoclonal antibodies such as rituximab also have antiangiogenic effects [27]. In the present study, we demonstrated that targeted therapy was a risk factor for stage 3 MRONJ and gefitinib was the most used targeted drug among MRONJ patients. Clinically, clinicians should give high attention to the oral hygiene and dental examination of patients and avoidance of invasive alveolar procedures in patients with combined administration of bisphosphonates and targeted drugs [24].

In agreement with other studies, the majority of MRONJ lesions occurred in the mandible [28, 29]. However, maxillary lesions were demonstrated to have a strong association with stage 3 MRONJ in the present study. According to the definition, exposed and necrotic bone extending beyond the region of alveolar bone involving inferior border of the mandible, sinus floor, or nasal base is identified as MRONJ stage 3 [7]. Generally, the distance between the maxillary alveolar ridge and sinus floor or nasal base is shorter than the distance between the mandibular alveolar ridge and inferior border. The maxillary bone is also more porous than the mandibular bone; thus, osteolysis in the maxilla can extend more easily. In addition, more vascularized maxilla deposits further bisphosphonates in the maxillary bone, which could result in severer MRONJ. Regarding pathological fracture and facial fistula, these complications were less likely occurred in the patients with maxillary osteonecrosis in this study. In other words, MRONJ that occurred in maxilla was more likely to develop into stage 3 by involving the maxillary sinus or nasal base.

Endocrine drugs and immunosuppressive drugs including GCs, thalidomide, and anti-PD-1 were found to be unrelated to stage 3 MRONJ. All patients with breast cancer and prostate cancer in this study had received endocrine drugs including bicalutamide and exemestane. The endocrine drug seems not to be an independent risk factor for the development of MRNOJ, while the GCs are a known risk factor for MRONJ [30]. Most immunosuppressive drugs were used in patients with multiple myeloma in this study. Only those patients who received GCs over 6 months were identified as use of GCs, which related to the negative result of this study possibly.

Time to onset (TTO) of ONJ, which is important for risk reduction and disease surveillance of MRONJ, is variably reported in different studies [17, 31]. In the present study, we found that the TTO of stage 3 MRONJ was longer than that of stage 2 MRONJ, but there was no statistical difference in TTO between two groups. In addition, bisphosphonates retain in the skeleton for years after discontinuance [32]. The longer the time from bisphosphonates discontinuance to the onset of MRONJ (dTTO) is, the less bisphosphonate is retained in the bone so that the jaw bone suffers less damage. Thus, in theory, the dTTO may be associated with the severity of MRONJ. Nevertheless, there was no favorable result of dTTO in relation to the stage 3 MRONJ in this study.

Whether serological biomarkers can be used for the diagnosis and prognosis of MRONJ is still controversial [33]. CTX, PINP, osteocalcin, calcitonin, PTH, 25-hydroxy vitamin D, Ca, Mg, P, and BAP are considered to be associated with bone metabolism. CTX, one of the serological bone turnover markers, is a research focus in recent years. Kwon et al. [34] reported that serum CTX had a significantly correlation with the severity of MRONJ. However, some studies showed insufficient evidence of MRONJ risk predicted by CTX [35, 36]. In the present study, no significant differences were found between the stage 2 and stage 3 MRONJ on all serological markers.

This study was blinded in that the researcher was unaware of the grouping to avoid bias. However, there still existed possible recall bias due to the retrospective nature of this study. Thus, prospective studies are necessary to provide more definitive scientific evidence about the risk factors for stage 3 MRONJ.

## 5. Conclusion

In conclusion, this is the first study recording comprehensive medication history of MRONJ patients and researching the risk factors for stage 3 MRONJ. The outcome of the present study justifies that chemotherapeutic agents and targeted drug use are probably risk factors for stage 3 MRONJ. In addition, the osteonecrosis in maxilla is more easily to develop into stage 3 MRONJ. Intense clinical observation is recommended in MRONJ patients with maxillary osteonecrosis and in those who concurrently received bisphosphonates, chemotherapeutic agents, and/or targeted drugs for their high risk of developing stage 3 MRONJ. Further studies are needed to provide more definitive scientific evidence.

## Figures and Tables

**Table 1:** 2019 MASCC/ISOO/ASCO staging system for MRONJ.

At risk	No symptoms and signs in patients who have been administered bone-modifying agents
Increased risk	No necrotic bone or fistulas that probe to bone but nonspecific symptoms, signs, and radiographic changes
Stage 1	Exposed and necrotic bone or fistulas that probe to bone in patients who are asymptomatic without evidence of infection
Stage 2	Exposed and necrotic bone or fistulas that probe to bone in patients who are symptomatic with or without purulent drainage
Stage 3	Exposed and necrotic bone or fistulas that probe to bone in patients who are symptomatic with infection, and one or more of the following: exposed and necrotic bone extending beyond the region of alveolar bone (i.e., inferior border and ramus in the mandible, maxillary sinus, and zygoma in the maxilla) resulting in pathologic fracture, extraoral fistula, oral antral/oral nasal communication, or osteolysis extending to the inferior border of the mandible of sinus floor

Abbreviations: MASCC/ISOO/ASCO: Multinational Association of Supportive Care in Cancer/International Society of Oral Oncology/American Society of Clinical Oncology; MRONJ: medication-related osteonecrosis of the jaw.

**Table 2:** Demographic and clinical characteristics of the two groups.

Variables	Group A: stage 2 (*N* = 31)		Group B: stage 3 (*N* = 29)		*P* values
	No.	%	No.	%	
Sex					
Male	16	51.6	16	55.2	0.782
Female	15	48.4	13	44.8	
Age (years)					
Range	61-89		60-85		
Mean ± SD	70.9 ± 10.0		69.1 ± 7.9		0.403
Smoking	12	38.7	15	51.7	0.311
Primary diseases					
Breast cancer	3	9.7	5	17.2	
Multiple myeloma	3	9.7	4	13.9	
Prostate cancer	10	32.3	2	6.9	
Pulmonary cancer	8	25.7	9	31.0	
Renal cancer	0	0	4	13.9	
Lymphoma	0	0	1	3.4	
Gastric carcinoma	1	3.2	0	0	
Rectum carcinoma	0	0	1	3.4	
Unknown cancer	0	0	1	3.4	
Osteoporosis	6	19.4	2	6.9	
General diseases					
Hypertension	15	48.4	12	41.4	0.586
Diabetes	8	25.8	10	34.5	0.464
Renal disease	2	6.5	5	17.2	0.247
Local factors					
Tooth extraction	21	67.7	22	75.9	0.485
Denture use	7	22.6	3	10.3	0.302
Periodontal disease	27	87.1	24	82.8	0.727
Chemotherapy	11	35.5	21	72.4	0.004^∗^
Targeted drugs	7	22.6	16	55.2	0.009^∗^
Endocrine drugs	13	41.9	7	24.1	0.144
Immunosuppressive drugs	4	12.9	8	27.6	0.155
Lesion location					
Mandible	25	80.6	18	62.1	0.111
Maxilla	6	19.4	15	51.7	0.009^∗^
Anterior region	4	12.9	5	17.2	0.727
Posterior region	27	87.1	24	82.8	0.727
TTO (months)					
Range	16-120		11-72		
Mean ± SD	28.7 ± 5.16		37.4 ± 19.9		0.144
dTTO (months)					
Range	0-36		0-21		
Mean ± SD	4.5 ± 8.5		3.8 ± 5.3		0.710

^∗^ *P* values were statistically significant. Abbreviations: SD: standard deviation; TTO: time from first using bisphosphonate to the onset of MRONJ; dTTO: time from last using bisphosphonate to the onset of MRONJ.

**Table 3:** The utilization of different medications between the two groups.

Medication	Group A: stage 2 (*N* = 31)		Group B: stage 3 (*N* = 29)		*P* values^1^
	*n* (%)	Mean ± SD	*n* (%)	Mean ± SD	
Bisphosphonates					
Zoledronic acid	25 (80.6)	35.0 ± 20.3	28 (96.9)	29.3 ± 18.2	0.285
Alendronate	6 (19.4)	75.0 ± 38.0	1 (3.1)	90.0	0.104
Routes					
IV	25 (80.6)		28 (96.9)		
Oral	6 (19.4)		1 (3.1)		
Chemotherapy					
Paclitaxel	0	0	3 (7.1)	7.3 ± 4.2	
Cisplatin	5 (27.7)	6.2 ± 1.5	11 (26.2)	11.2 ± 7.3	
Pemetrexed	6 (33.3)	5.5 ± 1.4	11 (26.2)	11.5 ± 7.2	
Tegafur/gimeracil/oteracil	2 (11.1)	10.0 ± 2.0	2 (4.8)	21.5 ± 20.5	
Gemcitabine	0	0	1 (2.4)	25.0	
Nedaplatin	1 (5.6)	5	0	0	
Cyclophosphamide (IV)	0	0	2 (4.8)	4.5 ± 0.5	
Cyclophosphamide (O)	1 (5.6)	24.0	2 (4.8)	25.0 ± 15.6	
Vindesine	0	0	3 (7.1)	5.3 ± 0.8	
Docetaxel	1 (5.6)	6.0	4 (9.5)	9.6 ± 2.3	
Bortezomib	2 (11.1)	67.5 ± 9.5	3 (7.1)	16.3 ± 2.6	
^2^Count	11 (35.4)	16.3 ± 21.4	21 (72.4)	14.6 ± 9.7	0.771
Targeted drugs					
Apatinib	3 (23.0)	19.0 ± 16.0	1 (4.5)	15.0	
Rituximab (time)	0	0	2 (9.2)	9.5 ± 7.5	
Erlotinib	1 (7.7)	50.0	0	0	
Icotinib	3 (23.1)	30.0 ± 6.5	2 (9.2)	28.0 ± 7.0	
Bevacizumab	1 (7.7)	24.0	2 (9.2)	22.0 ± 10.0	
Gefitinib	3 (23.1)	10.0 ± 2.0	5 (22.7)	40.3 ± 25.7	
Osimertinib	1 (7.7)	6.0	3 (13.6)	12.0 ± 3.0	
Sunitinib	0	0	3 (13.6)	45.7 ± 12	
Anlotinib	0	0	1 (4.5)	3.0	
Pazopanib	0	0	1 (4.5)	8.0	
Axitinib	0	0	1 (4.5)	5.0	
Sorafenib	0	0	1 (4.5)	72.0	
Afatinib	1 (7.7)	3.0	0	0	
^2^Count	7 (22.6)	37.9 ± 18.5	16 (55.2)	32.9 ± 23.4	0.629
Immunosuppressive drugs					
Thalidomide	2 (40.0)	66.0 + 42.0	3 (27.2)	23.3 ± 9.29	
GCs	2 (40.0)	NA	7 (63.6)	NA	
Anti-PD-1	1 (20.0)	1.0	1 (9.0)	3.0	

^1^ *P* values of medication duration or time were calculated. ^2^Count: the total number of patients administered with chemotherapy or targeted drugs and the total time or duration of these medications in the two groups were counted. ^∗^*P* values were statistically significant. The percentage of ^2^count (%) was the total number of patients receiving chemotherapy or targeted drugs divided by the total number of patients in different groups. SD: standard deviation; NA: not applicable.

**Table 4:** The laboratory examination results for the two groups.

Variables	Group A: stage 2 (*N* = 31)		Group B: stage 3 (*N* = 29)		*P* values
	Mean ± SD	Range	Mean ± SD	Range	
Serum Ca	2.12 ± 0.24	1.57-2.32	2.21 ± 0.11	2.02-2.38	0.277
Serum P	1.20 ± 0.14	0.99-1.46	1.16 ± 0.22	0.85-1.63	0.654
Serum Mg	0.90 ± 0.20	0.74-1.15	0.83 ± 0.12	0.78-1.38	0.323
PTH	45.29 ± 18.07	9.46-167.34	51.01 ± 41.68	22.54-80.42	0.720
CRP	26.91 ± 63.62	0.40-107.00	31.15 ± 34.23	1.28-184.00	0.844
Calcitonin	0.04 ± 0.02	0.02-0.68	0.03 ± 0.02	0.01-0.06	0.286
25-Hydroxy vitamin D	16.83 ± 9.77	8.17-36.60	17.85 ± 9.42	6.17-38.20	0.815
Osteocalcin	14.02 ± 3.77	7.31-41.00	15.60 ± 9.33	6.63-17.93	0.656
PINP	39.24 ± 20.30	11.98-136.10	47.64 ± 33.63	19.76-84.95	0.533
*β*-CTX	0.31 ± 0.14	0.12-1.34	0.40 ± 0.31	0.15-0.60	0.447
ALP	69 ± 22	34-93	111 ± 69	41-232	0.089

Abbreviations: SD: standard deviation; PTH: parathyroid hormone; CRP: C-reactive protein; PINP: carboxyl-terminal propeptide of type 1 procollagen; *β*-CTX: beta collagen degradation products; ALP: alkaline phosphatase.

**Table 5:** Results of multivariate logistic regression analysis.

Variable	*P* values	Crude OR	Adjusted OR	95% CI	
				Lower	Upper
Model 1					
Chemotherapy	0.044^∗^	4.77	3.43	1.03	11.38
Targeted drugs	0.040^∗^	4.22	3.69	1.06	12.80
Maxilla	0.026^∗^	4.46	4.26	1.19	15.23
Model 2					
Chemotherapy	0.013^∗^	—	4.26	1.35	13.40
Maxilla	0.028^∗^	—	3.91	1.16	13.19
Model 3					
Targeted drugs	0.013^∗^	—	4.58	1.38	15.16
Maxilla	0.012^∗^	—	4.85	1.42	16.61

The first model contained chemotherapy, targeted drugs, and maxillary lesions. The second model included chemotherapy and maxillary lesions. The third model contained targeted drugs and maxillary lesions. OR, 95% CI, and *P* values were calculated with the conditional multivariate logistic regression models according to likelihood ratio criteria. OR was mutually adjusted for variables in each model. ^∗^*P* values were statistically significant. Abbreviations: OR: odds ratio; 95% CI: 95% confidence interval.

## Data Availability

The data used to support the findings of this study are available from the corresponding author upon request.
